# Can We Replace Our Human Connection With Technology?

**DOI:** 10.1093/geroni/igab046.134

**Published:** 2021-12-17

**Authors:** Nicholas Cone, Jeongeun Lee

**Affiliations:** 1 Iowa State University, Iowa State University, Iowa, United States; 2 Iowa State University, Ames, Iowa, United States

## Abstract

The COVID-19 pandemic has led to social distancing protocols, subsequently increasing social isolation for older adults. The purpose of this study was to explore the relationship between social connectedness and mental health outcomes. Leveraging NHATS, a nationally representative study (n = 2,558, Mage = 79.20, SDage = 6.25), we examined the association between the method of social connectedness and mental health outcomes. Descriptive analyses revealed older adults are using various methods (e.g., in-person, phone, and video calls) to remain connected with their social networks during COVID-19. Findings from all of the linear regression analyses indicated phone or video calls are associated with negative affect, whereas in-person visits are associated with lower levels of negative affect. These findings suggest substituting in-person visits with video calls or phones may not be sufficient to relieve their loneliness and negative affect. Future studies should investigate this effect on physical or emotional health outcomes.

